# Childhood cancer care in Northern Tanzania: hospital infrastructure and provision of services

**DOI:** 10.3389/fped.2026.1785074

**Published:** 2026-04-15

**Authors:** Lexi Nussbaum, Yotham A. Gwanika, Prisca Joseph Stephano, Happiness D. Kajoka, Catherine Staton, Henry E. Rice, Blandina T. Mmbaga, Emily R. Smith, Pamela Espinoza, Esther Majaliwa

**Affiliations:** 1 Duke Global Health Institute, Durham, NC, United States; 2 Global Emergency Medicine Innovation and Implementation Research Center, Duke University, Durham, NC, United States; 3 Department of Oncology, Kilimanjaro Christian Medical Center, Moshi, Tanzania; 4 Department of Epidemiology and Biostatistics, School of Public Health, KCMC University, Moshi, Tanzania; 5 Department of Emergency Medicine, Duke University Medical Center, Durham, NC, United States; 6 Department of Surgery, Duke University School of Medicine, Durham, NC, United States; 7 Center for Global Surgery and Health Equity, Duke Global Health Institute, Duke University School of Medicine, Durham, NC, United States; 8 Kilimanjaro Clinical Research Institute, Moshi, Tanzania

**Keywords:** childhood cancer, health serivces, hospital infrastructure, Sub-Sarahan Africa, Tanzania

## Abstract

**Introduction:**

Survival rates for childhood cancer remain far lower in low- and middle-income countries (LMICs) compared to high-income countries (HICs). In Tanzania, challenges in cancer care for children are driven by shortages of trained providers, limited infrastructure, and constrained access to essential medications. This study evaluates pediatric oncology capacity and infrastructure in Northern Tanzania to identify system gaps and opportunities for improvement.

**Methods:**

A cross-sectional survey of capacity for pediatric cancer care was conducted at 25 hospitals across the Kilimanjaro, Arusha, Manyara, and Tanga regions in Tanzania. Facilities included health centers, district hospitals, regional hospitals, and one zonal referral hospital. Using a tool adapted from the International Society of Paediatric Oncology (SIOP) Global Mapping Survey, the World Health Organization Essential Medicines List, and the Global Initiative for Childhood Cancer, we collected data on hospital infrastructure from hospital leaders and staff. Key indicators included diagnostic imaging, pathology services, oncology workforce, medicine availability, treatment modalities, and cancer case volumes. Descriptive statistics were summarized using R.

**Results:**

Of the facilities surveyed, only one hospital (Kilimanjaro Christian Medical Centre) had a dedicated pediatric oncology ward and subspecialized staff. Although all facilities reported access to basic imaging such as x-ray and ultrasound, advanced imaging modalities (CT, MRI, specialized imaging) were confined to higher-level hospitals. Only 1 out of 25 hospitals offered pathology and pediatric surgical services. Among 20 essential pediatric oncology medicines assessed, only dexamethasone was universally available. District hospitals, despite serving the largest pediatric catchment areas and recording the highest admissions for children, lacked dedicated pediatric oncology wards.

**Discussion:**

Pediatric oncology services in Northern Tanzania are constrained by shortages in infrastructure, personnel, diagnostics, and medications. District hospitals have limited capacity to treat childhood cancer, resulting in critical delays in diagnosis and treatment. Strengthening infrastructure at the district level, creating efficient referral systems, and embedding pediatric oncology care into broader health systems may improve survival outcomes for children with cancer.

## 1 Introduction

Globally, about 400,000 children develop cancer each year (1). Low- and middle-income countries (LMICs) account for approximately 85% of cancer cases in children, with 5-year survival rates below 30% (2). This is in contrast to high-income countries (HICs), which report over 80% 5-year survival rates for children with cancer (3). Between 2020 and 2050, over 13 million pediatric cancer deaths are predicted. Without improvements in cancer care, 84% of these deaths are expected to occur in LMICs, emphasizing the need for stronger care and infrastructure (4).

Timely diagnosis and initiation of care is essential for improving outcomes in childhood cancer (5–9). Delays in care not only increase the risk of death but can also lead to lifelong disability, social stigma, and household impoverishment (10–12). Barriers in the timely diagnosis and cancer treatment in LMICs span throughout the entire continuum of care, from late presentation and diagnostic delays to treatment abandonment (13–16). Inadequate health infrastructure, especially at the primary care level, compounds these delays (17).

Tanzania, an LMIC in East Africa, exemplifies many of these challenges for cancer care due to a lack of trained healthcare personnel, limited diagnostic capacity, and poor treatment infrastructure (18, 19). Tanzania has fewer than 10 pediatric oncologists nationwide, of which the majority are located in the few tertiary-level hospitals (6). The country faces constrained access to cancer chemotherapy agents, with limited availability of several standard treatments that are readily accessible in HICs (16, 20). Prior work has found that these barriers are particularly challenging in the northern regions of Kilimanjaro, Arusha, Manyara, and Tanga; regions that make up more than 10% of Tanzania's population (5, 6, 9, 13, 15, 20, 21).

To improve childhood cancer outcomes in Tanzania, there is a need to identify infrastructure gaps to support long-term health system strengthening. Our aim was to evaluate pediatric oncology capacity and infrastructure in Northern Tanzania. Ultimately, this work should help identify system gaps and opportunities for improvement as well as contribute to a broader understanding of how to deliver equitable, timely, and high-quality cancer care for children in LMICs.

## 2 Methods

### 2.1 Setting

This study was conducted in Tanzania, a sub-Saharan country in East Africa. In 2024, Tanzania's GDP per capita was $1,185.7, classifying the country as a lower-middle income by World Bank criteria (22). The Northern Zone of the country includes four regions: Arusha, Kilimanjaro, Manyara, and Tanga with 633, 553, 361, and 675 total healthcare facilities, respectively. According to the 2022 Population and Housing Census, 6,804,733 people (14.1% of Tanzania's total population) live in these four regions (23). The health system in Tanzania is a tiered system where most families first access care at dispensaries or health centres before being referred to district, regional, or zonal hospitals. Specialized services, including cancer services, are concentrated at the zonal level, with limited diagnostic and treatment capacity at regional referral hospitals. Only a few hospitals provide specialized pediatric cancer care, including Muhimbili National Hospital (MNH) in the capital city of Dar es Salaam, which serves as the country's main referral centre for pediatric oncology, Ocean Road Cancer Centre (ORCI) also located in Dar es Salaam, Bugando Medical Centre (BMC) in Mwanza, Mbeya Regional Referral Hospital, and Kilimanjaro Christian Medical Centre (KCMC) in Moshi.

### 2.2 Participants

**Figure 1:**
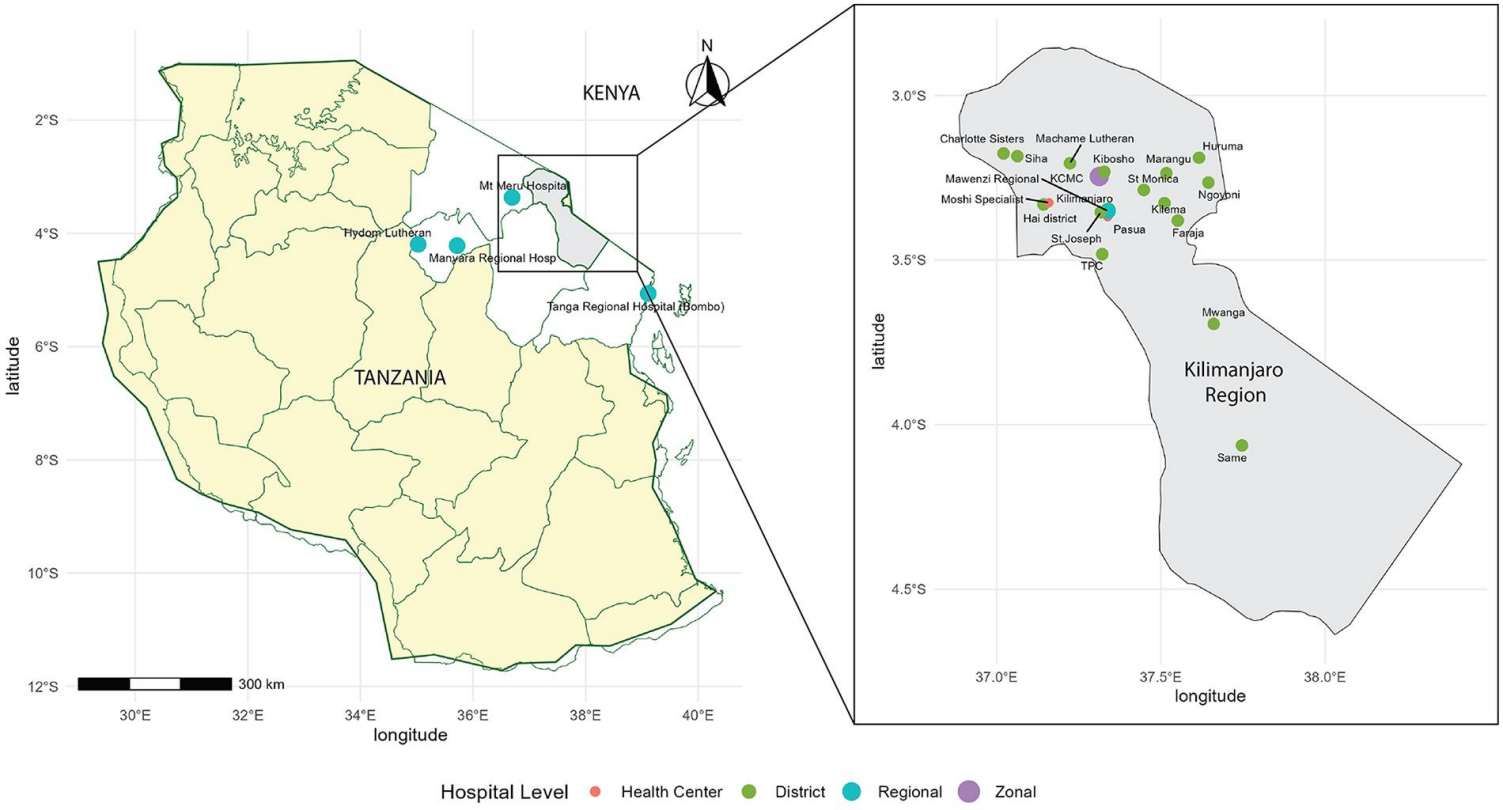
Map of surveyed hospitals in Northern Tanzania. Hai district: Hai District Hospital, Machame Lutheran Hospital, Moshi Specialist (District population size: 270,741).

In this study, we used a purposive sampling strategy based on the health facilities' role in providing pediatric surgical and cancer-related services within the Northern Zone. Within the Kilimanjaro region, we estimated that 125 healthcare facilities across all levels were capable of performing surgery based on national data (24). From these 125 facilities, we aimed to assess 100% of zonal, and regional referral hospitals and 25%—50% of district-level and lower-level facilities, resulting in a target sample of 14–22 facilities. To ensure comprehensive representation of referral pathways, 5 additional high-level facilities from the surrounding regions of Arusha, Manyara, and Tanga were included. In total, the study included 25 hospitals in the Northern Zone of Tanzania, representing different tiers of the healthcare system, including zonal hospitals, regional hospitals, district hospitals, and health centers (Figure 1). These included facilities reflect the hierarchical structure of the Tanzanian health system, incorporating district hospitals and regional referral hospitals across the Northern Zone. This distribution mirrors the typical referral pathway for pediatric cancer care and allows for an assessment of service capacity across different levels of care. Geographically, the sample consisted of 20 health facilities from the Kilimanjaro Region, 2 from the Arusha Region, 2 from the Manyara Region, and 1 from the Tanga Region. Of these, 9 were public hospitals and 16 were private hospitals. Hospitals that were fully private, or a private hospital with government partnership, were considered private in data collection. No facilities declined participation.

### 2.3 Hospital infrastructure, workforce, and capacity survey

Data was collected using several existing survey tools, which were integrated into a single tool for purposes of this study: The International Society of Paediatric Oncology (SIOP) Global Mapping of Pediatric Oncology Services Survey, the World Health Organization (WHO) Essential Pediatric Cancer Medicines List, and the six common tracer cancers of the Global Initiative for Childhood Cancer (GICC) (Supplementary Appendix S1). Our integrated survey tool aimed to assess the available hospital infrastructure, workforce, and capacity for cancer diagnosis in selected hospitals (1, 25, 26).

Hospital infrastructure data collected included the annual number of admissions and non-admitted patients, availability of inpatient hospital beds, catchment population, number of referred cases, availability of diagnostic imaging services, pathology services, patient admission and referral systems, essential cancer drugs, and availability of treatment modalities such as chemotherapy, surgery, and radiotherapy. Availability of medication was defined as routine availability at the facility, as reported by staff, reflecting whether medications are generally stocked. Additionally, facility details were recorded, including the presence of oncology wards, anesthesiology services, radiology services, (ultrasound, x-ray, bone and gallium scintigraphy, MRI, nuclear medicine services), pathological services, essential pediatric cancer medications, cancer volume (noting the number of diagnoses for conditions like acute lymphoblastic leukemia, acute myeloid leukemia, retinoblastoma, etc.), and method of payment for receiving childhood cancer services (25). Workforce information collected included the availability of oncology specialists, including physicians and nurses trained in pediatric oncology. General surgeons were defined as surgeons responsible for performing all surgical procedures across age groups and disease sites, including both adult and pediatric cases. Pediatric surgeons were defined as surgeons specifically trained to operate on pediatric populations while pediatric oncology surgeons were defined as surgeons who have the expertise in cancer-related surgical management for children. Visiting or shared surgical staff were counted if they routinely provided services at the facility during the study period. Childhood cancer descriptive information included the annual number of cancer diagnoses and treated patients for the six common tracer cancers of the GICC, as well as the total hospital admissions and outpatient visits (1). Summarized data on children who were 0 to 15 years old and were diagnosed and/or received cancer care at each facility were included. All patient-related data was de-identified and aggregated.

### 2.4 Data collection

Data collection took place between August 1, 2024, and February 28, 2025. Two data collectors trained in quantitative research conducted the fieldwork. Formal agreement to participate was obtained from each hospital. Hospital management directed the data collectors to key informants with adequate expertise in the oncology and pediatric departments to provide the required information for the study. Information was obtained from hospital leaders, division heads, consultants, junior faculty, nurses, social workers, volunteers, and other health workers. The data collected for purposes of this study were stored either in electronic health management systems [i.e., district health information software (DHIS2) and electronic management systems (eHMS) available at higher-level facilities] or in physical books primarily used in health centers [i.e., in Swahili: Mfumo wa Taarifa za Uendeshaji wa Huduma za Afya (MTUHA)]. Survey responses were initially collected on paper and subsequently entered into REDCap.

A major challenge encountered during data collection was the age categorization within the DHIS2 platform. Although our study used disaggregated data for children aged 0–15 years, the DHIS2 system only provided categories of 0–5 years and 5–60 years, which did not allow for isolation of the 5–15 year age group. To address this limitation, supplementary data were obtained from the MTUHA books, which record patient information in more detail without age groupings. This approach enabled retrieval of the required age-specific data and ensured the completeness of the dataset.

### 2.5 Data analysis

Data were analyzed using R Studio version 4.4.1. Descriptive statistics [i.e., medians, percentages, and interquartile range (IQRs)] were used to summarize responses. Where appropriate, data were stratified by facility level. There were no missing data. Map figure was created using R Studio version 4.4.1 (package: ggplot2) (27). This study complies with the Strengthening the Reporting of Observational Studies in Epidemiology (STROBE) Statement with the STROBE checklist attached (Supplementary Appendix S2).

### 2.6 Ethical considerations

Institutional review board (IRB) approval was granted from the Duke University (Protocol 00110763), the KCMC Institutional Review Board (Protocol 2,576), the Tanzania National Institute for Medical Research (NIMR: Protocol NIMR/HQ/R.8a/Vol.IX/4066). We also obtained ethical and administrative approvals from the National Institute for Medical Research (NIMR), the President's Office, Regional Administration and Local Government (TAMISEMI), and the respective District or Regional Medical Officers (DMO/RMO).

## 3 Results

### 3.1 Overall facility statistics

This study surveyed a total of 25 health facilities across Northern Tanzania. Within these 25 facilities, two (8%) were health centers, 17 (68%) were district hospitals, five (20%) were regional hospitals, and one (4%) was a zonal hospital.

### 3.2 Hospital characteristics by facility level

Facility characteristics varied widely across health system levels. Most district hospitals were private (82%, *n* = 14), most regional hospitals were public (80%, *n* = 4), and the zonal hospital (100%, *n* = 1) was private (Table 1). District hospitals recorded the highest number of pediatric admissions (>15,000 annually, median = 786.0), followed by regional (11,406, median = 1,813.0) and the one zonal hospital (6,317, median = 6,317.0). No facilities at the health center, district, or regional levels had dedicated pediatric oncology wards or beds, nor did they employ staff trained in pediatric oncology. Only the zonal hospital (KCMC) had a subspecialized pediatric oncology ward with 34 dedicated beds. This zonal facility employed two doctors trained in pediatric oncology, eight nurses who dedicated more than 75% of their time to pediatric cancer care, and one full-time pediatric oncology consultant. Although district and regional levels diagnosed cancer among children, only one zonal hospital (KCMC, *n* = 315) and one regional hospital treated children with cancer (Hydom Lutheran Hospital, *n* = 20). As for funding, only the zonal facility (KCMC) offered fully subsidized care from the government and/or donors, while all other facilities required full payment by families (Table 1).

**Table 1:** Hospital characteristics by facility level.

Characteristic	Health center (*N* = 2)	District (*N* = 17)	Regional (*N* = 5)	Zonal (*N* = 1)
General characteristics				
Facility type				
Public^+^	1 (50.0%)	3 (18.0%)	4 (80.0%)	0 (0.0%)
Private^+^	1 (50.0%)	14 (82.0%)	1 (20.0%)	1 (100.0%)
Inpatient hospital beds*	25 (12.5, 3.5)	328 (15.0, 17.0)	328 (63.0, 16.0)	170 (170.0, 0.0)
Pediatric oncology characteristics				
Dedicated oncology ward				
No pediatric oncology inpatient ward^+^	2 (100.0%)	17 (100.0%)	5 (100.0%)	0 (0.0%)
Subspecialized pediatric oncology ward^+^	0 (0.0%)	0 (0.0%)	0 (0.0%)	1 (100.0%)
Hospital beds dedicated to children with cancer in the hospital/oncology ward*	0 (0.0, 0.0)	0 (0.0, 0.0)	0 (0.0, 0.0)	34 (34.0, 0.0)
Healthcare personnel				
Doctors trained in pediatric oncology**	0	0	0	2
Nurses who care for children with cancer >75% of their time**	0	0	0	8
Full-time pediatric oncology consultants**	0	0	0	1
Part-time pediatric oncology consultants**	0	0	0	0
Cancer patient volume				
Children with cancer treated at the facility (annually)**	0	0	20	295
Children diagnosed with cancer at the facility (annually)**	0	35	64	83
Pediatric admissions annually				
Total number of pediatric admissions**	2,669	15,626	11,406	6,317
Median number pediatric admissions*	1,334.5 (834.25, 1,833.75)	768 (216, 1,197)	1,813 (1,350, 3,138)	6,317 (6,317, 6,317)
Median number of pediatric outpatients*	1,776 (1,264, 2,288)	4,853 (1,708, 6,720)	7,056 (4,186, 8,664)	4,728 (4,728, 4,728)
Median number of pediatric admissions under 5 years old in a year*	826 (539, 1,113)	436 (156, 798)	1,580 (1,360, 2,092)	4,462 (4,462, 4,462)
Median number of pediatric admissions 5–15 years old*	508.5 (296.25, 720.75)	184 (66, 327)	512 (453, 599)	1,855 (1,855, 1,855)
Method of payment				
Payment by family^+^	2 (100.0%)	17 (100.0%)	5 (100.0%)	0 (0.0%)
Fully subsidized by state/donors^+^	0 (0.0%)	0 (0.0%)	0 (0.0%)	1 (100.0%)

* [Median (IQR)] with the exception of the Zonal facility level category.

** *N.*

### 3.3 Availability of pathology and surgery services

Across all facilities surveyed, the availability of pathology and surgical services for pediatric cancer care was very limited. Of the facilities surveyed, only KCMC (4%), reported access to basic pathology services such as microscopy, hematoxylin and eosin staining, and cerebrospinal fluid cytology. More advanced diagnostic services, including immunohistochemistry panels, molecular pathology, cytogenetics, and genome sequencing were also limited to just KCMC (Table 2).

**Table 2:** Availability of pathology and surgery services .

Type of service	Available *N* (%)	Not available *N* (%)
Pathology services		
Microscope, H&E staining, CSF cytology	1 (4.0%)	24 (96.0%)
Limited immunohistochemistry panel, cytospin for CSF samples	1 (4.0%)	24 (96.0%)
Complete immunohistochemistry panel; molecular pathology and cytogenetics for most diseases; pediatric expertise necessary for specific diagnosis and staging; access to consultation with disease-specific expert pathologists at other centers	1 (4.0%)	24 (96.0%)
Research diagnostics, whole genome sequencing, and molecular pathology for all diseases	1 (4.0%)	24 (96.0%)
Hematopathology services		
Microscope, H&E staining, CSF cytology	1 (4.0%)	24 (96.0%)
Limited immunohistochemistry panel (disease-specific), flow cytometry, and cytogenetics are available most of the time	1 (4.0%)	24 (96.0%)
Flow cytometry of high quality; minimal residual disease testing; molecular pathology and cytogenetics; pediatric expertise; access to consultation with disease-specific expert pathologists at other centers	1 (4.0%)	24 (96.0%)
Research diagnostics, whole genome sequencing, and molecular pathology for all diseases	1 (4.0%)	24 (96.0%)
Pediatric surgery		
General surgeon; limited pediatric experience	2 (8.0%)	23 (92.0%)
Pediatric surgeon with limited oncology experience, oncology surgeon with limited pediatric experience	1 (4.0%)	24 (96.0%)
Pediatric oncology surgeon	0 (0.0%)	25 (100.0%)
Pediatric cancer surgeons with highly specialized disease-specific expertise	0 (0.0%)	25 (100.0%)
Surgical subspecialties		
Adult subspecialty surgeon	1 (4.0%)	24 (96.0%)
Some pediatric subspecialty surgeons	0 (0.0%)	25 (100.0%)
Full range of pediatric subspecialty surgeons	0 (0.0%)	25 (100.0%)
Pediatric subspecialty surgeons	0 (0.0%)	25 (100.0%)

### 3.4 Workforce

General surgeons were present in 8% (*n* = 2) of facilities, and only one facility reported having a pediatric or oncology surgeon with limited cross-disciplinary expertise. No facility had access to dedicated pediatric cancer surgeons with highly specialized disease-specific expertise. Pediatric surgical subspecialty services were entirely absent; almost all facilities (96%, *n* = 24) addressed pediatric surgical needs through adult subspecialty surgeons, and none had access to pediatric subspecialty surgeons (Table 2).

### 3.5 Availability of diagnostic imaging services

**Figure 2:**
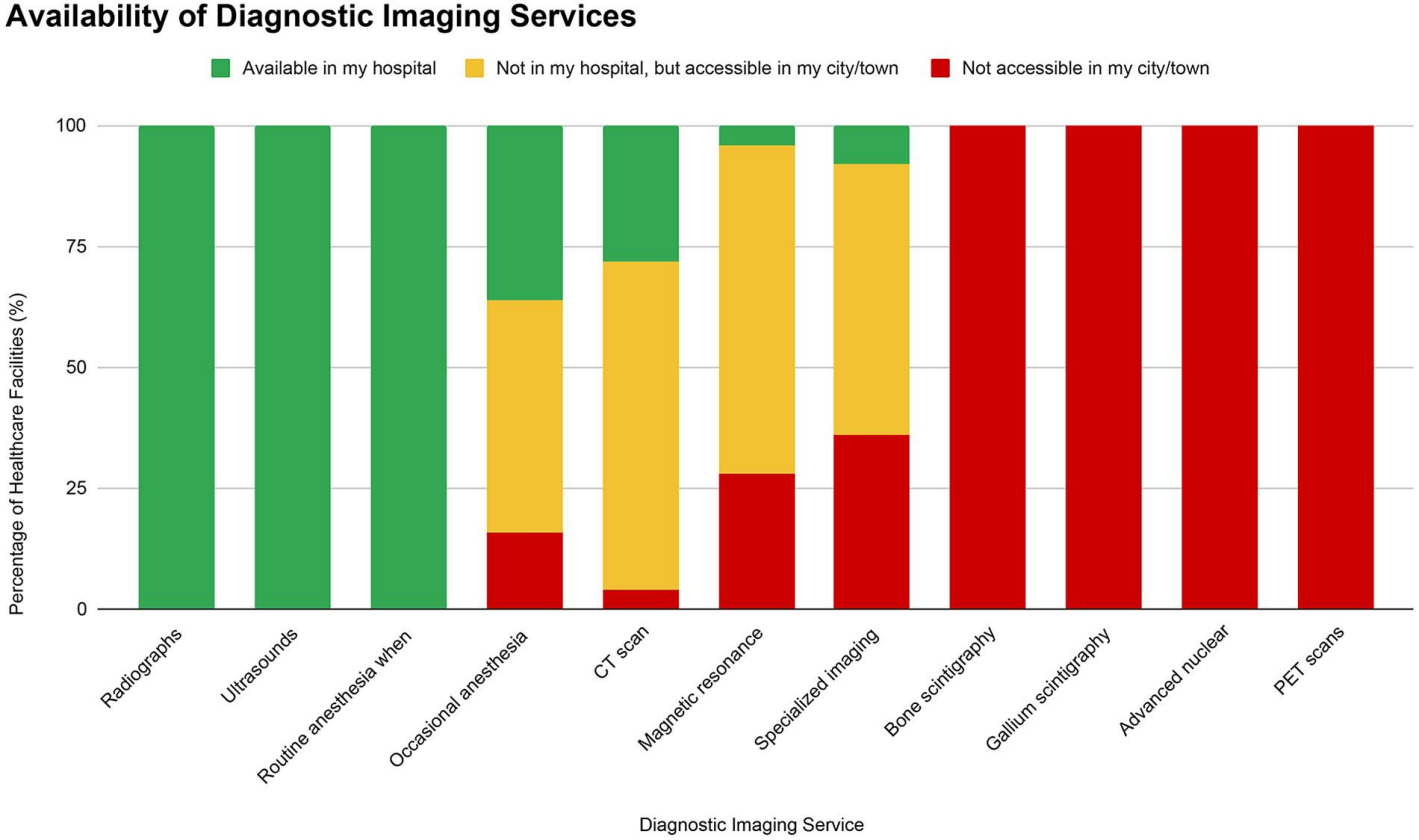
Availability of diagnostic imaging services.

Basic imaging modalities were widely accessible, with all healthcare facilities reporting availability of radiographs and ultrasounds, as well as anesthesia when needed. Most facilities reported availability of CT scans [either in-hospital (28%) or within the same city/town (68%)], magnetic resonance imaging (MRI) [either in-hospital (4%) or within the same city/town (68%)], and specialized imaging [either in-hospital (8%) or within the same city/town (56%)] (Figure 2).

### 3.6 Availability of essential pediatric cancer medicines

**Figure 3:**
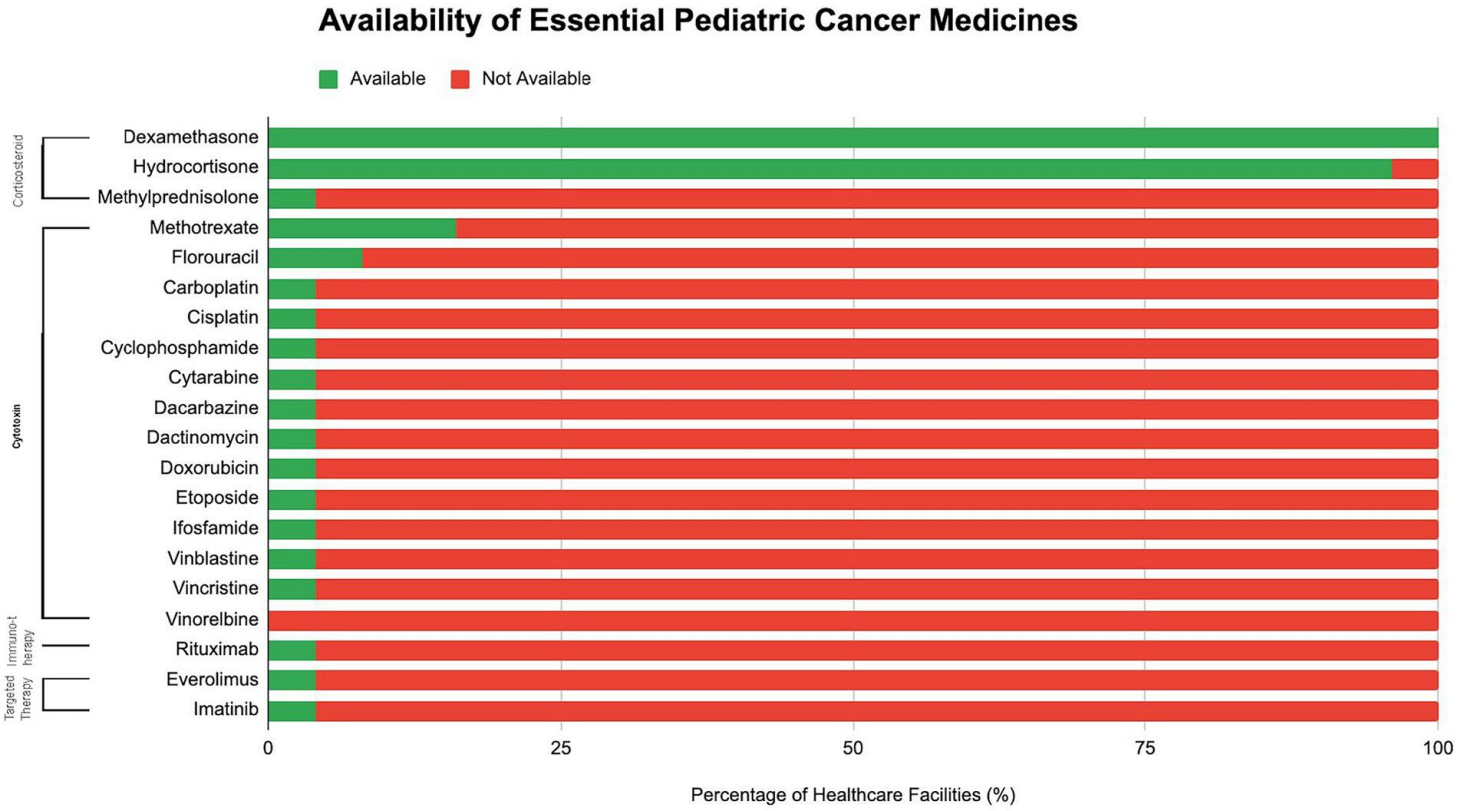
Availability of essential pediatric cancer medicines.

Among the 20 essential cancer medications assessed, only dexamethasone was universally available (100%, *n* = 25). Hydrocortisone was the second most accessible, present in 96% (*n* = 24) of facilities. All other chemotherapeutic and supportive agents were available in less than 25% of facilities, with 15 medications only available within one facility (4%, KCMC), and vinorelbine unavailable at all facilities (*n* = 0) (Figure 3).

### 3.7 Cancer diagnoses by facility level

**Figure 4:**
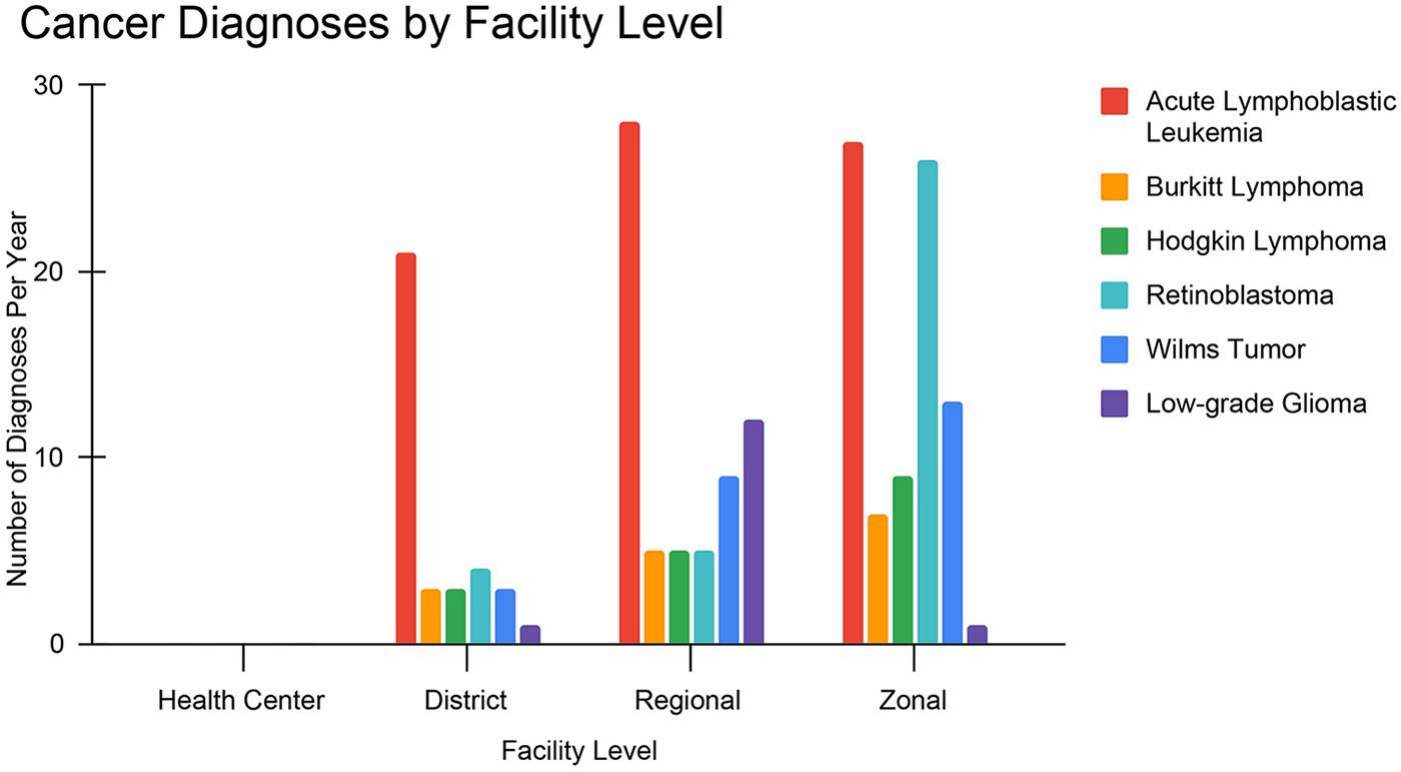
Cancer diagnoses by facility level.

Zonal hospitals diagnosed the highest number of pediatric cancer cases per year (*n* = 83). Acute lymphoblastic leukemia was the most frequently diagnosed cancer at all facility levels: at zonal hospitals (*n* = 27), regional hospitals (*n* = 28), and district hospitals (*n* = 21). Zonal facilities also reported the highest numbers of diagnoses for Wilms tumor (*n* = 14), Retinoblastoma (*n* = 26), Burkitt lymphoma (*n* = 7), and Hodgkin lymphoma (*n* = 9). Regional hospitals diagnosed the highest number of low-grade glioma cases (*n* = 12). Regional hospitals had moderate diagnosis volumes (*n* = 64) for a mix of cancer types, while district hospitals showed lower (*n* = 35) but broader diagnostic activity. Health centers reported no cancer diagnoses across all types (Figure 4).

## 4 Discussion

This study reveals disparities in pediatric oncology capacity in Tanzania across multiple health system levels. These structural gaps impact the capacity for early cancer detection, treatment, and overall care quality for pediatric patients with cancer. Although a limited number of higher-level facilities have specialized resources for cancer care, the pediatric oncology infrastructure across Northern Tanzania remains limited, with a minimal workforce and inconsistent access to diagnostic tools, medication, and specialized care.

Tanzania's referral process follows a stepwise model, where individuals are directed from lower-tier facilities to more advanced centers based on the severity and complexity of their condition (28). Although cancer care at all levels of facilities ideally should be improved, the district level seems to be the most urgent target for investment for children with cancer. District facilities serve the largest population catchment areas (almost 6 million people) and in turn report the highest number of pediatric admissions annually. District hospitals are often the first point of contact for sick children, including those with undiagnosed cancer. District levels lack any pediatric oncology specialists and have no inpatient ward, representing a critical missed opportunity in early diagnostics and care. In certain cancers, such as acute lymphoblastic leukemia, or Wilms' tumor, timely diagnosis and early initiation of therapy or surgery are strongly linked to survival outcomes (29). While district hospitals represent a critical leverage point within the referral system, strengthening pediatric capacity at this level requires consideration of feasibility and sustainability (7, 9). It is important to differentiate between two related, but distinct priorities for strengthening pediatric oncology systems: (1) improving early recognition and referral capacity at district facilities, and (2) decentralizing the delivery of chemotherapy or advanced diagnostics. A phased approach where early recognition and timely referral are prioritized can help strengthen the system without overextending already limited national oncology resources.

Current cancer referral systems in most LMICs, including Tanzania, often struggle with limited communication, transportation, and coordination between facilities (29–31). Where time is critical, especially in pediatric cancer, referral systems need to be streamlined to improve early diagnosis and treatment. One way to strengthen referral pathways is the strategic placement of imaging modalities. Basic imaging could be universally available at district hospitals to enable initial screening and early diagnoses, while CT and MRI capabilities may need to be concentrated in zonal facilities as they can serve broad catchment areas while also being cost-effective. Therefore, strengthening referral pathways from lower level hospitals with basic imaging to zonal hospitals with more advanced diagnostic and treatment capabilities, could improve the referral pathway for families with a child with cancer and reduce time delays from diagnosis to treatment initiation (32).

Our findings demonstrate that access to essential pediatric cancer medicines remains constrained. Similar challenges have been documented in other LMICs, where access to essential medicines and an adequately trained workforce remain critical bottlenecks in improving pediatric cancer outcomes (33). The WHO GICC emphasizes not only listing childhood cancer medicines on national essential medicines lists, but also ensuring their reliable procurement, distribution, and financial coverage (17). The inclusion of medicines on national lists has not consistently translated into availability at the facility level, largely due to weak procurement systems, fragmented supply chains, and insufficient forecasting capacity (34). Compounding these challenges is a shortage of trained health professionals, including pediatric oncologists, oncology pharmacists, and nurses, which limits the capacity to prescribe and administer available medicines appropriately (33). Aligning with GICC recommendations, strengthening pharmaceutical governance, ensuring sustainable financing for essential drugs, and investing in workforce training are indispensable to achieving equitable childhood cancer outcomes (17). Without simultaneous attention to both medicine access and workforce capacity, the promise of improved survival articulated in the GICC “CureAll” framework will remain out of reach in many LMIC settings.

Investing in hospital infrastructure can strengthen health systems while advancing care for children with cancer. Rather than purchasing expensive diagnostic equipment like CT or MRI machines solely for pediatric oncology, these tools can be integrated into broader pediatric health services including trauma, neurological disorders, infections, and congenital anomalies (7, 35). Strategic infrastructure development reinforces Universal Health Coverage by enabling efficient, technology-ready facilities that serve diverse health needs (36). Embedding cancer care within this wider system improves access, while also improving efficiency, equity, and long-term sustainability. Scaling up infrastructures in LMICs can have both substantial health and economic returns by preventing more deaths (17). Therefore, an approach that strengthens system-wide infrastructure maximizes care and benefits the entire health system.

### 4.1 Limitations

Our study has several limitations, many of which are common to hospital-based surveys and cross-sectional studies. We relied on key informants at each facility to obtain our data. With this, there is a risk the person interviewed might not have been the right person to answer all questions of the survey (particularly items not recorded in the health management systems). This might lead to over- or under-reporting of hospital infrastructure or availability of services. Our study only includes a subset of facilities who agreed to participate, which might lead to selection bias. However, we made an effort to include hospitals across all levels of the health system to capture the variability in infrastructure and provide a comprehensive assessment of childhood cancer care in the region. Due to our focus on infrastructure and availability of services, no outcome data was collected. For this reason, no conclusions can be made on the impact of hospital infrastructure on outcomes for children with cancer. There are also limitations within our data collection. First, the records did not identify how cancer diagnoses were confirmed (clinical vs. pathological confirmation) or referral patterns. Second, there was potential double counting between referring facilities and treatment centers, as we did not access individual data to track patients. However, the risk of double counting patients in the database is low since definitive diagnoses are mostly made at KCMC. Lastly, the cross-sectional nature of our study does not allow us to measure any improvements or changes after the data was collected.

## 5 Conclusion and recommendations

In conclusion, our findings reveal substantial service capacity and structural barriers for the provision of pediatric oncology in Northern Tanzania. Investment in hospital infrastructure, medicine availability, and workforce training, particularly at the district level, poses an opportunity to improve quality of care and reduce delays for children with cancer and their families. Strengthening infrastructure and scaling up referral systems at key entry points in the health system can significantly improve the provision of care, benefiting not only children with cancer but the entire population.

## Data Availability

The original contributions presented in the study are included in the article/Supplementary Material, further inquiries can be directed to the corresponding author.
