# Supplementary material for: Childhood cancer care in Northern Tanzania: hospital infrastructure and provision of services
Source: Front Pediatr. 2026 Apr 15;14:1785074. doi: 10.3389/fped.2026.1785074 (PMC13125148; doi:10.3389/fped.2026.1785074)
Supplement: Supplementary file 1 [file Supplementaryfile1.docx]

# Appendix 1: Hospital Assessment Tool

**

**

**Pediatric Cancer and Surgery: Hospital Assessment Tool**

| **GENERAL QUESTIONS** | | | | | | | | | | | | | | | |
| --- | --- | --- | --- | --- | --- | --- | --- | --- | --- | --- | --- | --- | --- | --- | --- |
| Questions developed by our team for this survey *(Appendix 12, K01 Surveys)* | | | | | | | | | | | | | | | |
|  | Level of facility: | Clinic/Polyclinic | Dispensary | | Health Center | | District | | Regional | Zonal | | | National | Specialized | |
|  | Location: | District |  | | Division | |  | | Ward |  | | | Village |  | |
|  | Which of the following terms best describe this healthcare facility? | Public | | | | Private | | | NGO/Mission/Charity | | | | University Hospital | | |
| SIOP Global Mapping of Pediatric Oncology Services Survey under “*General Information”* | | | | | | | | | | | | | | | |
|  | What is your position in your organization | Head of division | | Consultant | Junior faculty | | Nurse | Social Worker | | Volunteer | | | Patient | Parent | Other |
| **GENERAL INFRASTRUCTURE** | | | | | | | | | | | | | | | |
| WHO-PGSSC Surgical Assessment Tool (SAT) Hospital Walkthrough under “*Infrastructure: General Infrastructure”* | | | | | | | | | | | | | | | |
|  | Total number of admissions in a year | | | | | # | | | | | | | | | |
|  | Total number of outpatients seen in a year | | | | | # | | | | | | | | | |
|  | Total number of inpatient hospital beds | | | | | # | | | | | | | | | |
|  | Total number of surgical beds | | | | | # | | | | | | | | | |
|  | Total number of functioning operating rooms (major and minor) | | | | | # | | | | | | | | | |
|  | Total number of post-anesthesia care beds | | | | | # | | | | | | | | | |
|  | Total number of advanced care/ICU beds | | | | | # | | | | | | | | | |
|  | Total number of functional ventilators in the ICU | | | | | # | | | | | | | | | |
| Access and referral systems | | | | | | | | | | | | | | | |
|  | Total catchment population | | | | | # | | | | | | | | | |
|  | Number of pediatric surgical patients referred to a higher-level facility per month | | | | | # | | | | | | | | | |
|  | Number of pediatric oncology patients referred to a higher-level facility per month | | | | | # | | | | | | | | | |
| **FACILITY CHARACTERISTICS** | | | | | | | | | | | | | | | |
| Children’s Surgical Assessment Tool (CSAT): For Rwanda District Hospitals under *“Facility Characteristics”* | | | | | | | | | | | | | | | |
| For children <5 years old: | | | | | | | | | | | | | | | |
|  | Total number of pediatric inpatient admissions (>6 hours in non-emergency ward for children <5 in a year) | | | | | | | | | | | # | | | |
|  | Total number of pediatric Emergency Department visits for children <5 in a year | | | | | | | | | | | # | | | |
|  | Total number of children’s surgical admissions for children <5 in a year | | | | | | | | | | | # | | | |
| Questions developed by our team for this survey | | | | | | | | | | | | | | | |
|  | Total number of pediatric cancer admissions for children <5 in a year | | | | | | | | | | | # | | | |
| Children’s Surgical Assessment Tool (CSAT): For Rwanda District Hospitals under *“Facility Characteristics”* | | | | | | | | | | | | | | | |
| For children 5-15 years old: | | | | | | | | | | | | | | | |
|  | Total number of pediatric inpatient admissions (>6 hours in non-emergency ward) for children 5-15 years-old in a year | | | | | | | | | | | # | | | |
|  | Total number of pediatric Emergency Department visits for children 5-15 years old in a year | | | | | | | | | | | # | | | |
|  | Total number of children’s surgical admission for children 5-15 years old in a year | | | | | | | | | | | # | | | |
| Questions developed by our team for this survey | | | | | | | | | | | | | | | |
|  | Total number of pediatric cancer admissions for children 5-15 years old in a year | | | | | | | | | | | # | | | |
| SIOP Global Mapping of Pediatric Oncology Services Survey under “*Care Information*” | | | | | | | | | | | | | | | |
| What type of dedicated pediatric oncology programme does your hospital have?  □ Pilot project  □ Some basic oncology  □ Established oncology programme with most basic services and a few state-of-the-art services  □ Pediatric oncology programme with all essential services and most state-of-the-art services  □ Pediatric oncology programme, state-of-the-art services and some highly specialized services (e.g. proton beam radiation therapy, MIBG therapy, phase I studies)  □ Don’t know | | | | | | | | | | | | | | | |
| **FACILITY INFORMATION** | | | | | | | | | | | | | | | |
| SIOP Global Mapping of Pediatric Oncology Services Survey under “*Care Information*” | | | | | | | | | | | | | | | |
| Total number of beds dedicated/available to children with cancer in your hospital or pediatric oncology ward | | | | | | | | | | | # | | | | |

The following hospital assessment tool includes questions extracted from the following previously published resources: WHO-PGSSC Surgical Assessment Tool (SAT) Hospital Walkthrough, Children’s Surgical Assessment Tool (CSAT): For Rwanda District Hospitals, SIOP Global Mapping of Pediatric Oncology Services, WHO Essential Pediatric Cancer Medicines List, and Global Initiative for Childhood Cancer. The sources and modifications to the questions are outlined in red throughout the document.

| Children’s Surgical Assessment Tool (CSAT): For Rwanda District Hospitals under *“Facility Characteristics”* | |
| --- | --- |
| Total number of inpatient hospital beds dedicated to children’s surgery | # |
| Does your hospital have a pediatric recovery room? | Y or N |
| Total number of recovery room hospital beds dedicated to children’s surgery | # |
| Is oxygen available in the recovery room? | Y or N |
| Total number of neonatal unit/special baby care unit beds | # |
| Total number of pediatric ICU/advanced care beds | # |
| Access and referral systems: | |
| How many patients <5 years old per year do you refer to higher-level facilities for surgical interventions? | # |
| How many patients 5-15 years old per year do you refer to higher-level facilities for surgical interventions? | # |
| How far away is the nearest referral hospital (hours, by car) | hours |
| SIOP Global Mapping of Pediatric Oncology Services Survey under “*Care Information*” | |
| Do you have a dedicated pediatric oncology ward (inpatient ward)?  *Please choose the option that is most accurate in your setting*  □ No pediatric oncology inpatient ward  □ Area of the hospital where children with cancer are admitted when possible; frequent overflow to other wards; no fixed staff  □ Pediatric oncology inpatient ward available to most patients; limited fixed staff (e.g. oncology nurse permanently assigned)  □ Pediatric oncology inpatient ward separate from inpatient units for other patients; sufficient beds such that oncology patients rarely require admissions to other wards  □ Subspecialized pediatric oncology wards (e.g. transplant neuro-oncology, acute myeloid leukemia)  □ Don’t know | |

| Children’s Surgical Assessment Tool (CSAT): For Rwanda District Hospitals under *“Infrastructure”* | | | | | |
| --- | --- | --- | --- | --- | --- |
| **INFRASTRUCTURE** | | | | | |
| General Infrastructure - How often is this item available and functional? Choose (tick)  0- Unavailable (NOT AVAILABLE under any circumstances);  1- Inadequate (available to LESS THAN HALF of the time);  2- Limited (available to MORE THAN HALF, of the time but not all of the time)  Adequate (AVAILABLE all of the time without restrictions). | | Unavailable  (0) | Inadequate (1) | Limited  (2) | Adequate  (3) |
|  | 24-hour Emergency Unit able to receive pediatric patients |  |  |  |  |
| Pharmacy – product availability | | | | | |
|  | Pediatric malnutrition feeding program |  |  |  |  |
|  | Pediatric dosing cognitive aid or guide |  |  |  |  |
| Radiology & Pathology – service availability | | | | | |
|  | Does your hospital have a radiologist? | Y or N | | | |
|  | Does your hospital have an anesthesiologist who can perform sedation? | Y or N | | | |
|  | Enteral contrast material for bowel evaluation (barium or gastrografin) |  |  |  |  |
|  | Intravenous contrast material |  |  |  |  |
|  | Echocardiogram |  |  |  |  |
|  | Ultrasound |  |  |  |  |
|  | X-ray |  |  |  |  |
|  | Anatomic Pathology services |  |  |  |  |
| Blood Supply - availability | | | | | |
|  | Blood component transfusion |  |  |  |  |

| Children’s Surgical Assessment Tool (CSAT): For Rwanda District Hospitals under *“Service Delivery”* | | | | | | | | | | |
| --- | --- | --- | --- | --- | --- | --- | --- | --- | --- | --- |
| **SERVICE DELIVERY - SURGERY** | | | | | | | | | | |
| Rate adequacy as above  0 - Unavailable for Unavailable (**NOT AVAILABLE FOR ANYONE** who needs it);  1 - Inadequate (available to **LESS THAN HALF** of those who need it);  2 - Limited (available to **MORE THAN HALF,** but not to everyone who needs it); or  3 - Adequate (**PRESENT, AVAILIBLE** to almost everyone in need, and used when needed)  If less than adequate (rating 0, 1, or 2) then identify the barriers to access ----------------------------------------------------------------------------------------------> | | | **Access Barriers (Check all that apply)**  **Infrastructure** - physical space, equipment or materials.  **Absent** - has never has been present  **Broken** –resources present, but broken  **Personnel** - resource, service or function available, and staff trained, but limited availability at times (eg, night, weekend or holiday)  **Training** - No staff trained in using resource or performing function  **Stock out** - cannot be procured, or required equipment or supplies out of stock often due to poor stock management practices or procurement failures  **User fees** - available, but out-of-pocket payment requirement prevents delivery for some  **Other** - Other factors | | | | | | | |
| Deleted “Procedure Performed” Column | Total  procedures /  month  (Changed year to month) | Rate (0-3)  0-Unavailable  1-Inadequate  2-Limited  3-Adequate | **Infrastructure** | **Absent** | **Broken** | **Personnel** | **Training** | **Stock out** | **User fees** | **Other** |
| Suturing laceration |  |  |  |  |  |  |  |  |  |  |
| Drainage of superficial abscess |  |  |  |  |  |  |  |  |  |  |
| Wound debridement |  |  |  |  |  |  |  |  |  |  |
| Biopsy (tumor) |  |  |  |  |  |  |  |  |  |  |
| Male circumcision |  |  |  |  |  |  |  |  |  |  |
| Management of non-displaced fractures |  |  |  |  |  |  |  |  |  |  |
| Removal of foreign body from ear/nose |  |  |  |  |  |  |  |  |  |  |
| Support for emergency airway obstruction |  |  |  |  |  |  |  |  |  |  |
| Eyelid surgery for trachoma |  |  |  |  |  |  |  |  |  |  |
| Reduction of dislocation |  |  |  |  |  |  |  |  |  |  |
| Reduction and application of splint for non-displaced fractures |  |  |  |  |  |  |  |  |  |  |
| IV placement for neonates |  |  |  |  |  |  |  |  |  |  |
| **Procedures – Major in patients < 15 years old** |  |  |  |  |  |  |  |  |  |  |
| ***Children’s surgery*** |  |  |  |  |  |  |  |  |  |  |
| Appendectomy |  |  |  |  |  |  |  |  |  |  |
| Laparoscopy for typhoid peritonitis |  |  |  |  |  |  |  |  |  |  |
| Hernia/hydrocele repair |  |  |  |  |  |  |  |  |  |  |
| Non-operative reduction of intussusception |  |  |  |  |  |  |  |  |  |  |
| Operative reduction of intussusception |  |  |  |  |  |  |  |  |  |  |
| Bowel resection |  |  |  |  |  |  |  |  |  |  |
| Temporizing measures for gastroschisis and omphalocele (cover and rehydration) prior to referral |  |  |  |  |  |  |  |  |  |  |
| Rectal biopsy |  |  |  |  |  |  |  |  |  |  |
| Resection of solid abdominal masses |  |  |  |  |  |  |  |  |  |  |
| Creation of intestinal stomas |  |  |  |  |  |  |  |  |  |  |
| Emergency ostomies for imperforate anus |  |  |  |  |  |  |  |  |  |  |
| Closures of intestinal stomas |  |  |  |  |  |  |  |  |  |  |
| Resuscitation for pyloric stenosis |  |  |  |  |  |  |  |  |  |  |
| Catheterization / suprapubic cystostomy |  |  |  |  |  |  |  |  |  |  |
| Orchiopexy |  |  |  |  |  |  |  |  |  |  |
| Repair of testicular or ovarian torsion |  |  |  |  |  |  |  |  |  |  |
| Thyroidectomy (total or partial) |  |  |  |  |  |  |  |  |  |  |
| Drainage of septic arthritis / osteomyelitis |  |  |  |  |  |  |  |  |  |  |
| Repair of cleft lip and/or palate |  |  |  |  |  |  |  |  |  |  |
| ***Pediatric resuscitation and injury*** |  |  |  |  |  |  |  |  |  |  |
| Emergency Surgical airway (cricothyroidotomy) |  |  |  |  |  |  |  |  |  |  |
| Emergency Tube thoracostomy |  |  |  |  |  |  |  |  |  |  |
| Trauma Laparotomy |  |  |  |  |  |  |  |  |  |  |
| Conservative (non-operative) management for simple humeral fracture |  |  |  |  |  |  |  |  |  |  |
| Open reduction and internal fixation |  |  |  |  |  |  |  |  |  |  |
| Placement of pediatric external fixator |  |  |  |  |  |  |  |  |  |  |
| Emergency Escharotomy/fasciotomy |  |  |  |  |  |  |  |  |  |  |
| Contracture release |  |  |  |  |  |  |  |  |  |  |
| Amputations |  |  |  |  |  |  |  |  |  |  |
| Skin grafting |  |  |  |  |  |  |  |  |  |  |
| Emergency Burr hole |  |  |  |  |  |  |  |  |  |  |
| Emergency Craniotomy |  |  |  |  |  |  |  |  |  |  |
| **Procedures - Advanced (<15 years old)** |  |  |  |  |  |  |  |  |  |  |
| Surgery for neonatal acute abdomen |  |  |  |  |  |  |  |  |  |  |
| Repair of club foot |  |  |  |  |  |  |  |  |  |  |
| Placement and management of external ventricular drain |  |  |  |  |  |  |  |  |  |  |
| Definitive repair of non-complicated anorectal malformation or Hirschsprung’s Disease |  |  |  |  |  |  |  |  |  |  |

| SIOP Global Mapping of Pediatric Oncology Services Survey under “*Diagnostic imaging services*” | | | | | | |
| --- | --- | --- | --- | --- | --- | --- |
| **SERVICE DELIVERY - CANCER** | | | | | | |
| **Diagnostic imaging services** | | | | | | |
|  | **Available in my hospital** | **NOT in my hospital, but accessible in my city/town** | | **Not accessible in my city/town** | | **Don’t know** |
| Radiographs |  |  | |  | |  |
| Ultrasounds |  |  | |  | |  |
| CT scan |  |  | |  | |  |
| Bone scintigraphy |  |  | |  | |  |
| Gallium scintigraphy |  |  | |  | |  |
| Occasional availability of anesthesia when needed |  |  | |  | |  |
| Magnetic resonance imaging |  |  | |  | |  |
| PET scan available to most patients |  |  | |  | |  |
| Routine availability of anesthesia when needed |  |  | |  | |  |
| Specialized imaging |  |  | |  | |  |
| Advanced nuclear medicine applications (e.g. Metaiodobenzylguanidine [MIBG] scanning): |  |  | |  | |  |
| SIOP Global Mapping of Pediatric Oncology Services Survey under “*Pathology services*” | | | | | | |
| **Pathology services** | | | | | | |
| Does your hospital or pediatric oncology ward have access to adequate pathology services?  *Please choose the option that is most accurate in your setting*  □ No  □ Microscope, H&E staining, CSF cytology  □ Limited immunohistochemistry panel (disease-specific), cytospin for CSF samples  □ Complete immunohistochemistry panel ; molecular pathology and cytogenetics for most diseases; pediatric expertise necessary for specific diagnosis and staging; access to consultation with disease-specific expert pathologists at other centers  □ Research diagnostics, whole genome sequencing, molecular pathology for all diseases  □ Don’t know | | | | | | |
| Does your hospital or pediatric oncology ward have access to hematopathology services?  *Please choose the option that is most accurate in your setting*  □ No  □ Microscope, H&E staining, CSF cytology  □ Limited immunohistochemistry panel (disease-specific), flow cytometry and cytogenetics available most of the time  □ Flow cytometry of high-quality; minimal residual disease testing; molecular pathology and cytogenetics; pediatric expertise; access to consultation with disease specific expert pathologists at other centers  □ Research diagnostics, whole genome sequencing, molecular pathology for all diseases  □ Don’t know | | | | | | |
| SIOP Global Mapping of Pediatric Oncology Services Survey under “*Other services”* | | | | | | |
| **Other services** | | | | | | |
| Do you have an active pediatric oncology clinical research programme at your hospital or pediatric oncology ward | □ Yes □ No □ Don’t know | | | | | |
| Do children with cancer have access to radiotherapy services at your hospital or pediatric oncology ward? | □ Yes □ No □ Don’t know | | | | | |
| Do children with cancer have access to pediatric surgery at your hospital or pediatric oncology ward?  *Please choose the option that is most accurate in your setting*  □ General surgeon; limited pediatric experience  □ Pediatric surgeon with limited oncology experience, oncology surgeon with limited pediatric experience  □ Pediatric oncology surgeon  □ Pediatric cancer surgeons with highly specialized disease-specific expertise  □ No  □ Don’t know | | | | | | |
| Do children with cancer have access to surgical subspecialities relevant to oncology at your hospital or pediatric oncology ward?  *Please choose the option that is most accurate in your setting*  □ Adult subspecialty surgeon (neurosurgeon, orthopedic surgeon, ophthalmologist, other)  □ Some pediatric subspecialty surgeons (neurosurgeon, orthopedic surgeon, ophthalmologist, other)  □ Full range of pediatric subspecialty surgeons (neurosurgeon, orthopedic surgeon, ophthalmologist, other)  □ Pediatric subspeciality surgeons with highly specialized disease-specific expertise  □ No  □ Don’t know | | | | | | |
| Do you have established regular pediatric oncology multidisciplinary team meetings at your hospital or pediatric oncology ward?  *Please choose the option that is most accurate in your setting*  □ Ad hoc meetings for special cases  □ Routinely scheduled meetings with reasonable attendance  □ Real-time discussion of all complex cases to guide important care decisions  □ Pediatric patients discussed as part of combined multidisciplinary teams  □ Pediatric patients discussed in dedicated pediatric oncology multidisciplinary teams  □ Incorporation of molecular and genetic expertise in meetings; cancer-specific multidisciplinary meetings like a CNS tumor or sarcoma meeting  □ No  □ Don’t know | | | | | | |
| SIOP Global Mapping of Pediatric Oncology Services Survey under “*Non profit organizations in your area”* | | | | | | |
| **Non-profit organizations in your area** | | | | | | |
| Please check all the services that are provided by non-profit organizations in your area to you or children with cancer and their families:  □ Transport  □ Accommodation  □ Food  □ Other financial assistance  □ Specialist certification  □ Advise institutions on cancer control policies negotiate working conditions  □ Fund raising  □ Public awareness for cancer  □ Research assistance  □ Other | | | | | | |
| *WHO Essential Pediatric Cancer Medicines List* | | | | | | |
| **Essential Pediatric Cancer Medicines** | | | | | | |
|  | Available | | Not Available | | Don’t know | |
| Carboplatin |  | |  | |  | |
| Cisplatin |  | |  | |  | |
| Cyclophosphamide |  | |  | |  | |
| Cytarabine |  | |  | |  | |
| Dacarbazine |  | |  | |  | |
| Dactinomycin |  | |  | |  | |
| Dexamethasone |  | |  | |  | |
| Doxorubicin |  | |  | |  | |
| Etoposide |  | |  | |  | |
| Everolimus |  | |  | |  | |
| Florouracil |  | |  | |  | |
| Hydrocortisone |  | |  | |  | |
| Ifosfamide |  | |  | |  | |
| Imatinib |  | |  | |  | |
| Methotrexate |  | |  | |  | |
| Methylprednisolone |  | |  | |  | |
| Rituximab |  | |  | |  | |
| Vinorelbine |  | |  | |  | |
| Vinblastine |  | |  | |  | |
| Vincristine |  | |  | |  | |

| **Surgical Volume** | | |
| --- | --- | --- |
| WHO-PGSSC Surgical Assessment Tool (SAT) Hospital Walkthrough under “*Service Delivery: Surgical Volume”* | | |
|  | Number of laparotomies performed per month *(changed “per year” to “per month”)* | # |
|  | Number of C-sections performed per month *(changed “per year” to “per month”)* | # |
|  | Number of open fracture repairs performed per moth *(changed “per year” to “per month”)* | # |
| Children’s Surgical Assessment Tool (CSAT): For Rwanda District Hospitals under *“Surgical Volume”* | | |
|  | Number of pediatric laparotomies performed per month *(changed “per year” to “per month”)* | # |
|  | Number of elective hernia repairs per month *(changed “per year” to “per month”)* | # |
|  | Number of neonatal (< 1 month age) stomas performed per month *(changed “per year” to “per month”)* | # |
|  | Number of surgical repairs of pediatric open fractures performed per month *(changed “per year” to “per month”)* | # |
|  | Total number of procedures performed in pediatric patients per month *(changed “per year” to “per month”)* | # |
|  | Percent of surgery cases that were emergent or urgent (non-elective) cases | % |
|  | At what age do you start performing elective hydrocele/hernia repair? | Years |
| **Cancer Volume** | | |
| SIOP Global Mapping of Pediatric Oncology Services Survey under “*Care Information*” | | |
|  | Number of children with cancer treated each year at hospital or pediatric oncology ward | # |
| Six common, tracer cancers for the Global Initiative for Childhood Cancer | | |
|  | Number of children diagnosed with acute lymphoblastic leukemia (a blood cancer) per year | # |
|  | Number of children diagnosed with Burkitt lymphoma (a fast-growing lymph gland cancer) per year | # |
|  | Number of children diagnosed with Hodgkin lymphoma (a lymph gland cancer) per year | # |
|  | Number of children diagnosed with Retinoblastoma (a childhood eye cancer) per year | # |
|  | Number of children diagnosed with Wilms tumor (a childhood kidney cancer) per year | # |
|  | Number of children diagnosed with low-grade glioma (a brain cancer) per year | # |

| Children’s Surgical Assessment Tool (CSAT): For Rwanda District Hospitals under *“Quality and Safety”* | | | | | | | | | | |
| --- | --- | --- | --- | --- | --- | --- | --- | --- | --- | --- |
| **Quality and Safety** | | | | | | | | | | |
| Does your institution use electronic medical records? | | | | | | □ Yes □ No | | | | |
| Does your institution have a trauma registry that includes pediatric trauma? | | | | | | □ Yes □ No | | | | |
| What is the highest ASA class of children operated at your institution? (ASA I = healthy patient, ASA II = patient with mild systemic disease, ASA III = patient with severe systemic disease, ASA IV = patient with severe systemic disease that is a constant threat to life) | | | | | | □1 □2 □3 □4 | | | | |
| Is the WHO Surgical Safety Checklist used in the operating rooms for pediatric patients? | | | | | | □ Never □ Less than half the time  □ More than half the time □ All the time | | | | |
| Is pulse oximetry used in the operating rooms for pediatric patients? | | | | | | □ Never □ Less than half the time  □ More than half the time □ All the time | | | | |
|  | | | | | | | | | | |
| Children’s Surgical Assessment Tool (CSAT): For Rwanda District Hospitals under *“Operating Equipment and Supplies”* | | | | | | | | | | |
| **Operating Equipment and Supplies – How often is the following equipment available and functional for surgery?** | | | | | | | | | | |
| Rate adequacy as above  0 - Unavailable for Unavailable (**NOT AVAILABLE FOR ANYONE** who needs it);  1 - Inadequate (available to **LESS THAN HALF** of those who need it);  2 - Limited (available to **MORE THAN HALF,** but not to everyone who needs it); or  3 - Adequate (**PRESENT, AVAILIBLE** to almost everyone in need, and used when needed)  If less than adequate (rating 0, 1, or 2) then identify the barriers to access -------------------------------------------------------------------------------------------> | | **Access Barriers (Check all that apply)**    **Infrastructure** - physical space, equipment or materials.  **Absent** - has never has been present  **Broken** –resources present, but broken  **Personnel** - resource, service or function available, and staff trained, but limited availability at times (eg, night, weekend or holiday)  **Training** - No staff trained in using resource or performing function  **Stock out** - cannot be procured, or required equipment or supplies out of stock often due to poor stock management practices or procurement failures  **User fees** - available, but out-of-pocket payment requirement prevents delivery for some  **Other** - Other factors | | | | | | | | |
|  | Rate (0-3)  0-Unavailable  1-Inadequate  2-Limited  3-Adequate | **Infrastructure** | **Absent** | **Broken** | **Personnel** | | **Training** | **Stock out** | **User fees** | **Other** |
| Functional anesthesia machines in the ORs (added question by our team) |  |  |  |  |  | |  |  |  |  |
| Functional anesthesia machines with pediatric breathing system |  |  |  |  |  | |  |  |  |  |
| Pediatric oropharyngeal airway (000-4) |  |  |  |  |  | |  |  |  |  |
| Pediatric endotracheal tubes (2.5 - 6 mm) |  |  |  |  |  | |  |  |  |  |
| Pediatric laryngoscope(Miller ≤ 2 or Macintosh ≤ 3) |  |  |  |  |  | |  |  |  |  |
| Pediatric facemask bag valve or Ambu bag (< 550ml bag with < size 3 mask) |  |  |  |  |  | |  |  |  |  |
| Pediatric difficult airway kit (LMA) |  |  |  |  |  | |  |  |  |  |
| Pediatric Magill forceps |  |  |  |  |  | |  |  |  |  |
| Pediatric blood pressure monitor or cuff |  |  |  |  |  | |  |  |  |  |
| Pediatric pulse oximetry |  |  |  |  |  | |  |  |  |  |
| Pediatric nasogastric Tube ( <12 Fr) |  |  |  |  |  | |  |  |  |  |
| Pediatric chest tubes (< 20 Fr) |  |  |  |  |  | |  |  |  |  |
| Pediatric surgical instruments |  |  |  |  |  | |  |  |  |  |
| Pediatric urinary catheters (<12 Fr) |  |  |  |  |  | |  |  |  |  |
| Pediatric central lines (< 7 Fr or 12 cm |  |  |  |  |  | |  |  |  |  |
| Sutures (3.0, 4.0, 5.0, 6.0) |  |  |  |  |  | |  |  |  |  |
| Minimally invasive equipment (laparoscopy, arthroscopy, and thoracoscopy) |  |  |  |  |  | |  |  |  |  |
| Capnography (exp. CO2 measurement) |  |  |  |  |  | |  |  |  |  |
| Pre-formed intestinal silos or pre-formed/readily available silos created from plastic bags |  |  |  |  |  | |  |  |  |  |

| **WORKFORCE** | | | |
| --- | --- | --- | --- |
| Children’s Surgical Assessment Tool (CSAT): For Rwanda District Hospitals under *“Workforce”* | | | |
| **Children's Surgery Provider Density** | | | |
| **Providers** | **Full Time** | | **Part Time** |
| Number of qualified general surgeons with pediatric exposure (trained surgeons with expertise in pediatric or neonatal surgery, but without formal subspecialty pediatric surgical training) | # | | # |
| Number of qualified general surgeons with pediatric exposure (trained surgeons with expertise in pediatric or neonatal surgery, but without formal subspecialty pediatric surgical training) | # | | # |
| Number of qualified general pediatric surgeons (trained surgeons with formal specialized training in children’s surgery of ≥ 1 year) | # | | # |
| Number of general doctors providing pediatric surgery (general practitioners without formal surgical training) | # | | # |
| Number of non-physicians providing pediatric procedure (non- physicians health care professional who performs procedures independently without formal training in surgery). These procedures include: circumcision, reduction of closed fractures, small wound repair, debridement, minor skin grafts. |  | |  |
| Number of qualified anesthesiologists |  | |  |
| Number of qualified pediatric anesthesiologists (trained anesthesiologists with formal specialization) |  | |  |
| Number of general doctors providing pediatric anesthesia (general practitioners without formal anesthesiology training) |  | |  |
| Number of non-physicians providing pediatric anesthesia (non- physicians health care professionals who perform pediatric anesthesia independently without formal training in anesthesia) |  | |  |
| Number of nurses with training or exposure in pediatric surgery, who treat only children |  | |  |
| Number of visiting or consultant physicians who are involved in pediatric surgical care (visiting at least 1x/week for at least 1 year).  Type ______________ Type ______________ Type ______________  # ____________ # ____________ # ____________ |  | |  |
| Please identify pediatric specialists that are available at your hospital *(indicate number in brackets)*  *□ general pediatrician ( ) □ hematologist ( )*  *□ cardiac surgeon ( ) □ nephrologist ( )*  *□ dental surgeon ( ) □ neurologist ( )*  *□ neurosurgeon ( ) □ respiratory physician ( )*  *□ ophthalmologist ( ) □ neonatologist ( )*  *□ orthopedic surgeon( ) □ cardiologist ( )*  *□ otorhinolaryngologist (ENT) ( ) □ endocrinologist ( )*  *□ plastic surgeon ( ) □ physiotherapist ( )*  *□ urologist ( )* |  | |  |
| Please identify staff members that are present in your hospital (indicate number in brackets) |  | |  |
| □ radiographer ( )  □ pediatric intensive care nurse ( ) □ radiologist ( ) □ pathologist ( ) □ neonatal nurse ( ) □ speech therapist ( ) □ oncologist treating children ( ) □ qualified nutritionist ( ) □ audiologist ( ) □ ophthalmology technician ( ) |  | |  |
| SIOP Global Mapping of Pediatric Oncology Services Survey under “*Pediatric oncology staff*” | | | |
| **Pediatric oncology staff** | | | |
| How many FULL-TIME pediatric oncology consultants (pediatricians with fellowship training in pediatric oncology) are there at  your hospital or pediatric oncology ward? | | # | |
| How many PART-TIME pediatric oncology consultants (pediatricians with fellowship training in pediatric oncology) are there at  your hospital or pediatric oncology ward? | | # | |
| How many doctors who are trained in pediatric oncology, care for children with cancer at your hospital or pediatric oncology ward? | | # | |
| How many nurses care for children with cancer the majority (>75%) of their time at your hospital or pediatric oncology ward? | | # | |
| **FINANCING** | | |  |
| Children’s Surgical Assessment Tool (CSAT): For Rwanda District Hospitals under *“Financing”* | | |  |
| **Health financing and accounting** | | |  |
| What percentage of children coming to this hospital have health insurance | □ None □ Fewer than half □ More than half □ All | |  |
| Nationally, is there government-sponsored health insurance/financing for children? | □ Yes □ No | |  |
| On average, what percentage pediatric surgical costs for patients are covered by insurance? | % | |  |
| Please choose the statement that is most true regarding payment of care in your setting:  SIOP Global Mapping of Pediatric Oncology Services Survey under “*Care Information*” | Childhood cancer treatment is:  □ fully subsidized by state/donors  □ partially subsidized by state/donors  □ paid for by the family of the patient | |  |
| **Budget Allocation** | | |  |
| Annual hospital budget allotted to children’s surgery and anesthesia | Actual amount: % of total budget: | |  |
| **Cost: average total inpatient cost for a patient for…** | Cost | % Out of Pocket |  |
| pediatric hernia/hydrocele repair |  |  |  |
| pediatric fracture management |  |  |  |
| pediatric laparotomy |  |  |  |
| Pediatric appendectomy |  |  |  |
| **Average non-medical cost to a patient for a pediatric hernia/hydrocele repair**  (consider transport, lodging, food for patient and family) | Cost | |  |

# Appendix 2: STROBE Checklist

STROBE Statement—Checklist of items that should be included in reports of ***cross-sectional studies***

|  | **Item No** | **Recommendation** | **Page No** |
| --- | --- | --- | --- |
| **Title and abstract** | 1 | (*a*) Indicate the study’s design with a commonly used term in the title or the abstract | 2 |
|  |  | (*b*) Provide in the abstract an informative and balanced summary of what was done and what was found | 2 |
| **Introduction** | | | |
| Background/rationale | 2 | Explain the scientific background and rationale for the investigation being reported | 3 |
| Objectives | 3 | State specific objectives, including any prespecified hypotheses | 3 |
| **Methods** | | | |
| Study design | 4 | Present key elements of study design early in the paper | 4-5 |
| Setting | 5 | Describe the setting, locations, and relevant dates, including periods of recruitment, exposure, follow-up, and data collection | 4 |
| Participants | 6 | (*a*) Give the eligibility criteria, and the sources and methods of selection of participants | 4 |
| Variables | 7 | Clearly define all outcomes, exposures, predictors, potential confounders, and effect modifiers. Give diagnostic criteria, if applicable | 4-5 |
| Data sources/ measurement | 8* | For each variable of interest, give sources of data and details of methods of assessment (measurement). Describe comparability of assessment methods if there is more than one group | *5* |
| Bias | 9 | Describe any efforts to address potential sources of bias | NA |
| Study size | 10 | Explain how the study size was arrived at | 4 |
| Quantitative variables | 11 | Explain how quantitative variables were handled in the analyses. If applicable, describe which groupings were chosen and why | 5 |
| Statistical methods | 12 | (*a*) Describe all statistical methods, including those used to control for confounding | 5 |
|  |  | (*b*) Describe any methods used to examine subgroups and interactions | 5 |
|  |  | (*c*) Explain how missing data were addressed | 5 |
|  |  | (*d*) If applicable, describe analytical methods taking account of sampling strategy | NA |
|  |  | (*e*) Describe any sensitivity analyses | NA |
| **Results** | | | |
| Participants | 13* | (a) Report numbers of individuals at each stage of study—eg numbers potentially eligible, examined for eligibility, confirmed eligible, included in the study, completing follow-up, and analysed | 6 |
|  |  | (b) Give reasons for non-participation at each stage | NA |
|  |  | (c) Consider use of a flow diagram | NA |
| Descriptive data | 14* | (a) Give characteristics of study participants (eg demographic, clinical, social) and information on exposures and potential confounders | 6-7 |
|  |  | (b) Indicate number of participants with missing data for each variable of interest | 6-7 |
| Outcome data | 15* | Report numbers of outcome events or summary measures | 6-7 |
| Main results | 16 | (*a*) Give unadjusted estimates and, if applicable, confounder-adjusted estimates and their precision (eg, 95% confidence interval). Make clear which confounders were adjusted for and why they were included | 6-7 |
|  |  | (*b*) Report category boundaries when continuous variables were categorized | 6-7 |
|  |  | (*c*) If relevant, consider translating estimates of relative risk into absolute risk for a meaningful time period | NA |
| Other analyses | 17 | Report other analyses done—eg analyses of subgroups and interactions, and sensitivity analyses | NA |
| **Discussion** | | | |
| Key results | 18 | Summarise key results with reference to study objectives | 8-9 |
| Limitations | 19 | Discuss limitations of the study, taking into account sources of potential bias or imprecision. Discuss both direction and magnitude of any potential bias | 9 |
| Interpretation | 20 | Give a cautious overall interpretation of results considering objectives, limitations, multiplicity of analyses, results from similar studies, and other relevant evidence | 8-9 |
| Generalisability | 21 | Discuss the generalisability (external validity) of the study results | 8-9 |
| **Other information** | | | |
| Funding | 22 | Give the source of funding and the role of the funders for the present study and, if applicable, for the original study on which the present article is based | 9 |

*Give information separately for exposed and unexposed groups.

**Note:** An Explanation and Elaboration article discusses each checklist item and gives methodological background and published examples of transparent reporting. The STROBE checklist is best used in conjunction with this article (freely available on the Web sites of PLoS Medicine at http://www.plosmedicine.org/, Annals of Internal Medicine at http://www.annals.org/, and Epidemiology at http://www.epidem.com/). Information on the STROBE Initiative is available at www.strobe-statement.org.
